# Kinetic study of NADPH activation using ubiquinone-rhodol fluorescent probe and an Ir^III^-complex promoter at the cell interior

**DOI:** 10.1039/d3ra05412h

**Published:** 2023-11-20

**Authors:** Hirokazu Komatsu, Nadiia Velychkivska, Anastasiia B. Shatan, Yutaka Shindo, Kotaro Oka, Katsuhiko Ariga, Jonathan P. Hill, Jan Labuta

**Affiliations:** a Research Center for Materials Nanoarchitectonics (MANA), National Institute for Materials Science (NIMS) 1-1 Namiki Tsukuba Ibaraki 305-0044 Japan Labuta.Jan@nims.go.jp; b Institute of Macromolecular Chemistry, Czech Academy of Sciences Heyrovsky Sq. 2 Prague 6 162 06 Czech Republic; c Department of Bioscience and Informatics, Faculty of Science and Technology, Keio University 3-14-1 Hiyoshi, Kohoku Yokohama Kanagawa 223-8522 Japan; d Waseda Research Institute for Science and Engineering, Waseda University 2-2 Wakamatsucho, Shinjuku-ku Tokyo 162-8480 Japan; e Graduate Institute of Medicine, College of Medicine, Kaohsiung Medical University Kaohsiung City 80708 Taiwan; f Department of Advanced Materials Science, Graduate School of Frontier Sciences, The University of Tokyo 5-1-5 Kashiwanoha, Kashiwa Chiba 277-8561 Japan

## Abstract

Nicotine adenine dinucleotide derivatives NADH and NADPH are intimately involved in energy and electron transport within cells. The fluorescent ubiquinone-rhodol (Q-Rh) probe is used for NADPH activation monitoring. Q-Rh reacts with NADPH yielding its quenched hydroquinone-rhodol (H_2_Q-Rh) form with concurrent NADPH activation (*i.e.* NADP^+^ formation). NADPH activation can be enhanced by the addition of an Ir^III^-complex (*i.e.* [(η^5^-C_5_Me_5_)Ir(phen)(H_2_O)]^2+^) as a promoter. The rate of the Q-Rh fluorescence quenching process is proportional to the NADPH activation rate, which can be used to monitor NADPH. Experiments were performed in phosphate-buffered saline (PBS) solution and on HeLa cell cultures to analyze the kinetics of Q-Rh reduction and the influence of the Ir^III^-complex promoter on the activation of NADPH (in PBS) and of other intracellular reducing agents (in HeLa cells). There is a substantial increase in Q-Rh reduction rate inside HeLa cells especially after the addition of Ir^III^-complex promoter. This increase is partly due to a leakage process (caused by Ir^III^-complex-induced downstream processes which result in cell membrane disintegration) but also involves the nonspecific activation of other intracellular reducing agents, including NADH, FADH_2_, FMNH_2_ or GSH. In the presence only of Q-Rh, the activation rate of intracellular reducing agents is 2 to 8 times faster in HeLa cells than in PBS solution. When both Q-Rh and Ir^III^-complex are present, the rate of the Ir^III^-complex catalyzed reduction reaction is 7 to 23 times more rapid in HeLa cells. Concentration- and time-dependent fluorescence attenuation of Q-Rh with third-order reaction kinetics (reasonably approximated as pseudo-first-order in Q-Rh) has been observed and modelled. This reaction and its kinetics present an example of “bioparallel chemistry”, where the activation of a molecule can trigger a unique chemical process. This approach stands in contrast to the conventional concept of “bioorthogonal chemistry”, which refers to chemical reactions that occur without disrupting native biological processes.

## Abstract 2

We study kinetics of the reaction of fluorescent ubiquinone-rhodol (Q-Rh) with NADPH leading to the quenched reduced hydroquinone-rhodol (H_2_Q-Rh) form with simultaneous NADPH activation. Activation is enhanced by addition of Ir^III^-complex promoter.

## Graphical abstract

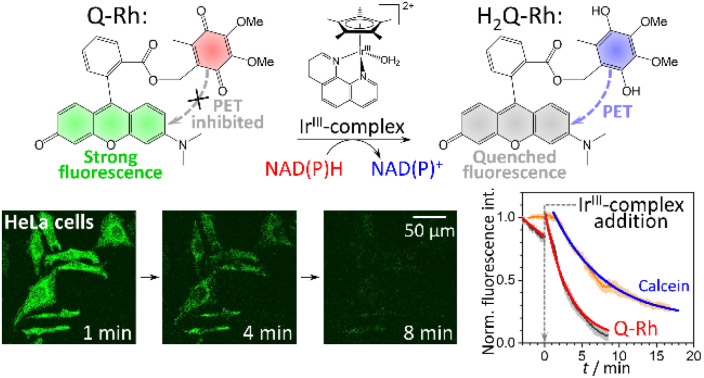

## 1 Introduction

**Fig. 1:**
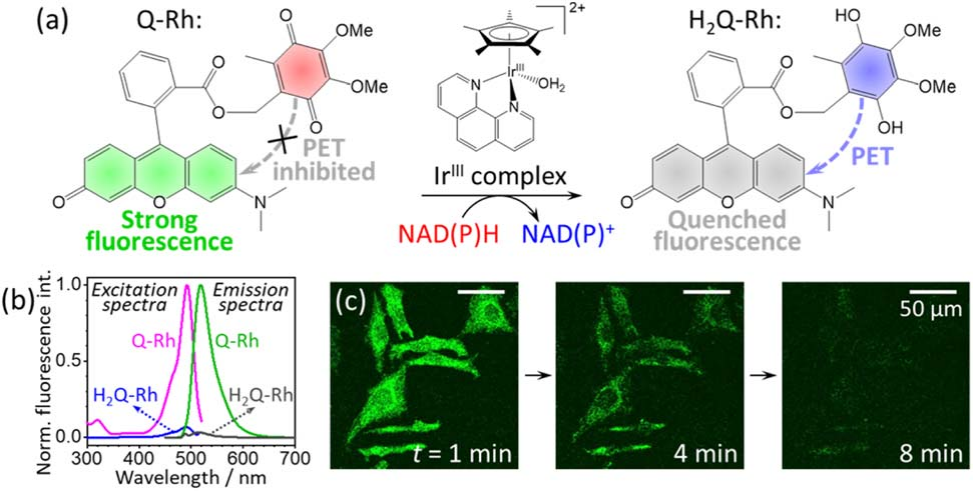
(a) Reduction reaction of ubiquinone-rhodol (Q-Rh) to hydroquinone-rhodol (H_2_Q-Rh) by nicotinamide adenine dinucleotide derivatives (NAD(P)H) in the presence of [(η^5^-C_5_Me_5_)Ir(phen)(H_2_O)]^2+^ complex (Ir^III^-complex). (b) Normalized fluorescence excitation (at *λ*_em_ = 520 nm) and fluorescence emission spectra (at *λ*_ex_ = 488 nm) of Q-Rh and its reduced (by Na_2_S_2_O_4_) form H_2_Q-Rh in phosphate-buffered saline (PBS) at pH = 7.4 and 25 °C. (c) Micrographs of fluorescence quenching of Q-Rh stained (0.01 mM) HeLa cells at 1, 4 and 8 min following the addition of Ir^III^-complex.

The bioorthogonal chemistry approach is used to examine biomolecules in their native environment using chemical reactions that do not interfere with the biological processes.^1–5^ In contrast to biorthogonal chemistry, “bioparallel chemistry” is attributed to chemical reactions involving artificial molecules interacting with native biological processes, and has been introduced by Komatsu *et al.* in 2014.^6^ The intracellular activation of acetyl coenzyme A (acetyl-CoA) by tributylphosphine (PBu_3_), and its fluorescent detection, is considered the first successful example of the bioparallel chemistry concept.^7^ In a further study, an artificial reaction promoter (PBu_3_) was used to control ATP concentration and acetylation of mitochondrial proteins.^8^ These results effectively illustrate that novel artificial reaction promoters can be excellent candidates for intracellular imaging and are promising for the modulation of cellular functions. In 2013, Sadler and coworkers reported the reduction of quinone by reduced coenzyme NADH involving a cyclopentadienyl–Ir^III^ catalyst complex in aqueous media.^9,10^ Subsequently, in 2014, Komatsu *et al.* reported the use of a fluorescent ubiquinone-rhodol (Q-Rh) conjugate containing a biocompatible rhodol fluorophore^11^ for intracellular activation and imaging of nicotinamide adenine dinucleotide (NAD) derivatives NADH and NADPH. Both NADH and NADPH act as electron transporters in living cells and play a crucial role in metabolism.^12,13^ Here, a kinetic study has been undertaken to understand the reaction mechanism of NADPH in the presence of Q-Rh fluorescent dye and an Ir^III^-complex (*i.e.* [(η^5^-C_5_Me_5_)Ir(phen)(H_2_O)]^2+^) promoter (Fig. 1a). Information regarding rates of chemical reactions (including in the biological system), reaction order, and rate-determining steps^14,15^ is essential for the further development of quinone reduction processes that mimic the action of reductases, such as NADH ubiquinone oxidoreductase,^16^ NADH cytochrome-*b*_5_,^17^ and NADPH cytochrome P-450 reductase.^18^

In this study, we present an analysis of the Ir^III^-complex catalyzed reduction of Q-Rh by NADPH yielding hydroquinone-rhodol (H_2_Q-Rh) and activated NADP^+^ (Fig. 1a). This reaction is important as an one of the leading examples of bioparallel chemical processes in living organisms.

## 2 Results and discussion

### 2.1 Kinetics in phosphate-buffered saline (PBS) solution

The Q-Rh fluorescent probe (synthesized according to Komatsu *et al.*^6^) has absorbance and fluorescence maxima at 492 and 518 nm, respectively (Fig. 1b), with a fluorescence quantum yield of 0.73 in phosphate-buffered saline (PBS) at pH = 7.4. The typical fluorescence lifetime of rhodol-type dyes is around 4 ns in aqueous phosphate buffer.^19^ The reduced form of Q-Rh (*i.e.* H_2_Q-Rh) obtained using sodium dithionite (Na_2_S_2_O_4_)^20^ has significantly attenuated UV-vis absorption and its fluorescence emission is strongly quenched involving photoinduced-electron transfer (PET) mechanisms, as shown in Fig. 1b. Micrographs illustrating the time dependence of fluorescence emission of Q-Rh at the interior of HeLa cells following addition of the Ir^III^-complex are shown in Fig. 1c. Also in this study, we elucidate the kinetics behind the time dependence of fluorescence emission intensity. However, first we will consider the normal kinetics of Q-Rh to H_2_Q-Rh conversion under simple conditions (in PBS, pH = 7.4) in the presence of NADPH and in the absence/presence of the Ir^III^-complex promotor (which activates NADPH).

The following equation describes the reduction reaction of Q-Rh by NADPH.1
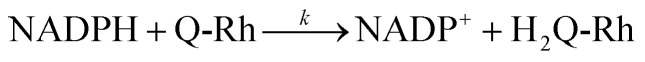
It should be noted that the overall reaction in eqn (1) consists of the following two processes.^21^2
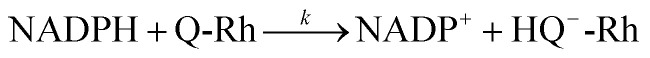
3
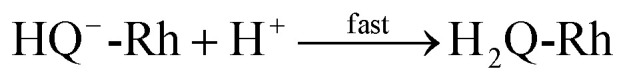
The reaction in eqn (2) describes the direct hydride transfer leading to the formation of hydroquinone anion (HQ^−^-Rh) and is followed by the reaction in eqn (3), where hydroquinone H_2_Q-Rh is formed due to the rapid protonation of HQ^−^-Rh by H^+^ from the medium.^21^ This indicates that the reaction shown in eqn (2) is the rate-limiting step. Therefore, the reaction rate constant, *k*, for the overall reaction (eqn (1)) is the same as that for the reaction in eqn (2).

In the presence of the Ir^III^-complex reaction promoter, the following reversible association process between NADPH and Ir^III^-complex is assumed to occur.4
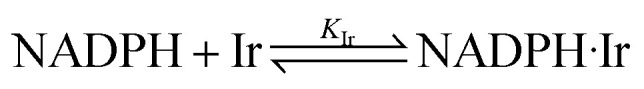
where Ir represents the promoter Ir^III^-complex, NADPH·Ir is the complexed form of NADPH reactant with the Ir^III^-complex, and *K*_Ir_ = [NADPH·Ir]/([NADPH][Ir]) is the equilibrium association constant (square brackets denote concentrations of the species). Kinetics of the reaction in the presence of reactant-promoter NADPH·Ir complex is governed by the following reaction.5
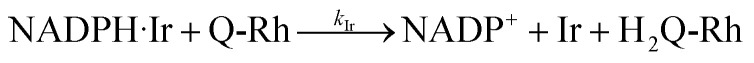
where *k*_Ir_ is the reaction rate constant of this process (*i.e.* the process in which the NADPH·Ir complex reduces Q-Rh). Thus, in the presence of Ir^III^-complex promoter, all three processes described by the reactions in eqn (1), (4) and (5) are simultaneous. The solution of these kinetics is done using the following approach. The reaction progress was monitored by fluorescence emission from the Q-Rh probe (at 518 nm). The reactions shown in eqn (1) and (5) lead to the following differential rate equation for the decrease of Q-Rh concentration.6
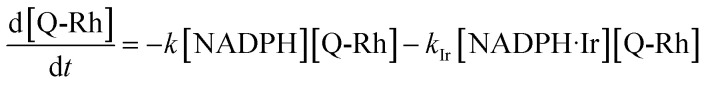
Substituting the [NADPH·Ir] term using the definition of *K*_Ir_ (given in the context of the reaction in eqn (4)) followed by rearrangement yields a differential rate equation for Q-Rh in the following form.7
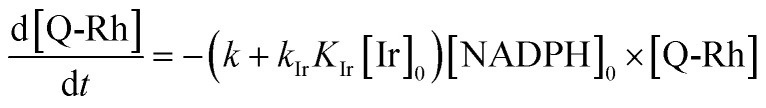
In this study, the concentration of Q-Rh fluorescent probe is always significantly lower than those of the NADPH reactant and Ir^III^-complex promoter. Therefore, they can be assumed constant, *i.e.* [NADPH] = [NADPH]_0_ and [Ir] = [Ir]_0_, where [NADPH]_0_ and [Ir]_0_ are initial concentrations of NADPH and Ir^III^-complex promoter, respectively. This situation is denoted by ‘0’ subscripts in eqn (7). The above assumptions reduce the initially third-order rate eqn (7) (*i.e.*, first-order in [Ir], [NADPH] and [Q-Rh]) to pseudo-first-order in Q-Rh concentration with a pseudo-first-order rate constant

8 *k*′ = (*k* + *k*_Ir_*K*_Ir_[Ir]_0_)[NADPH]_0_.

The eqn (7) can then be solved analytically in the form of eqn (9).^14^

9 [Q-Rh] = [Q-Rh]_0_exp(−*k*′*t*)

where [Q-Rh]_0_ is the initial concentration of Q-Rh probe. The time dependency of hydroquinone H_2_Q-Rh concentration can be readily derived considering the mass balance equation [Q-Rh]_0_ = [Q-Rh] + [H_2_Q-Rh].

10 [H_2_Q-Rh] = [Q-Rh]_0_(1 − exp(−*k*′*t*))

Taking into account that the fluorescence emission of reacted quenched H_2_Q-Rh (at 518 nm) is 1/30 (=*q*) of the Q-Rh starting fluorescence intensity (*i.e. ca.* 97% quenching efficiency) due to the operation of PET mechanism (see Fig. 1b and Experimental section for more details), we can assume that the time dependence of the normalized fluorescence intensity *I*_n_(*t*) at 518 nm is proportional to [Q-Rh] + *q*[H_2_Q-Rh]. Then the resulting *I*_n_(*t*) can be expressed as eqn (11), where normalization means that *I*_n_(*t* = 0) = 1.

11 *I*_n_(*t*) = *q* + (1 − *q*)exp(−*k*′*t*)

**Fig. 2:**
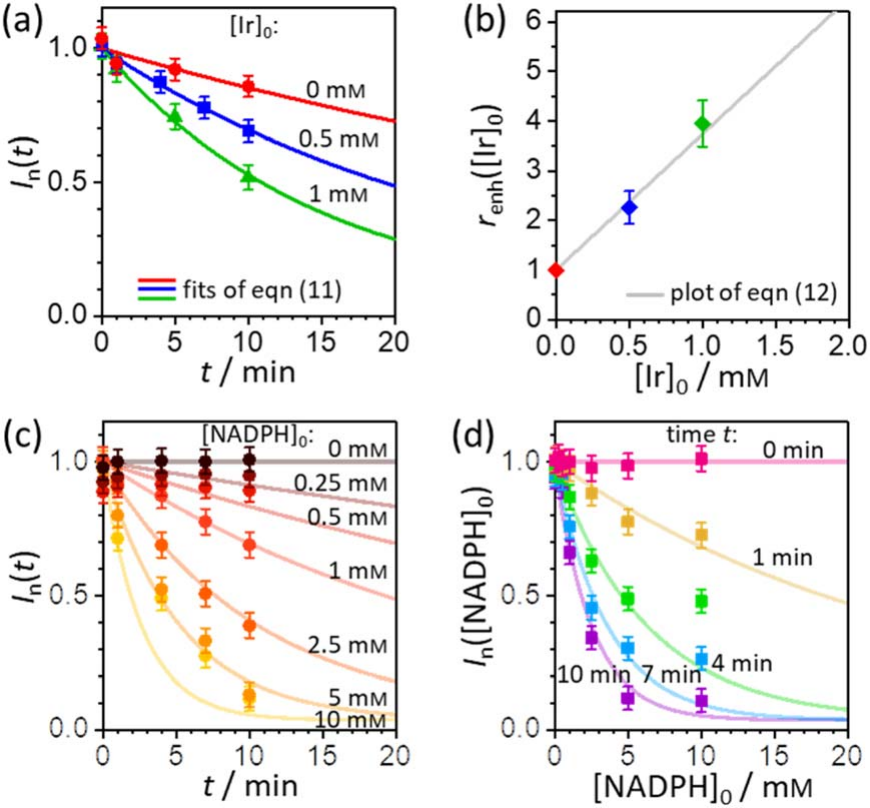
(a) Plot of Q-Rh time-dependent normalized fluorescence intensity *I*_n_(*t*) (at 520 nm) as a function of Ir^III^-complex promoter concentration: [Ir]_0_ = 0 mM (red data points), 0.5 mM (blue data points), and 1 mM (green data points). Experiments were performed in PBS solution (100 mM, pH = 7.4, 25 °C) at constant [NADPH]_0_ = 1 mM and [Q-Rh]_0_ = 0.01 mM. Solid lines are the best fits using eqn (11). (b) Plot of enhancement ratio *r*_enh_ as a function of Ir^III^-complex promoter concentration. The data points are obtained from the evaluation of *r*_enh_ independently from each experiment in (a). The grey solid line is a plot of eqn (12). (c) Plot of time-dependent Q-Rh normalized fluorescent intensity *I*_n_(*t*) (at 520 nm) recorded after Q-Rh addition ([Q-Rh]_0_ = 0.01 mM) at *t* = 0 min into the PBS solutions (100 mM, pH = 7.4, 25 °C) containing constant Ir^III^-complex concentration ([Ir]_0_ = 0.5 mM) and varying NADPH concentration ([NADPH]_0_ = 0–10 mM). Solid lines are plots (not fits) of eqn (11) with *k* and *k*_Ir_*K*_Ir_ values from Table 1 and *t* is an independent variable. (d) Plot of Q-Rh normalized fluorescent intensity *I*_n_([NADPH]_0_) (at 520 nm) as a function of [NADPH]_0_ concentration recorded at various times (*t* = 0–10 min) after Q-Rh addition ([Q-Rh]_0_ = 0.01 mM) into the PBS solutions (100 mM, pH = 7.4, 25 °C) containing constant Ir^III^-complex concentration ([Ir]_0_ = 0.5 mM). Solid lines are plots (not fits) of eqn (11) with *k* and *k*_Ir_*K*_Ir_ values from Table 1 and [NADPH]_0_ as an independent variable.

In order to extract the kinetic parameters, the time-dependent normalized fluorescence of Q-Rh (0.01 mM) with NADPH (1 mM) was measured at different Ir^III^-complex promoter concentrations in PBS solution, as shown in Fig. 2a. In the absence of Ir^III^-complex, the pseudo-first-order rate constant reduces to *k*′ = *k*[NADPH]_0_, and fitting of eqn (11) to experimental data (Fig. 2a) yields a value of the reaction rate constant *k* = 0.28 ± 0.06 M^−1^ s^−1^ describing the kinetics in eqn (1). After the addition of Ir^III^-complex (0.5 and 1 mM) into the solution (and using already-known *k*), the fitting procedure (Fig. 2a) further yields the value of the product *k*_Ir_*K*_Ir_ = 764 ± 85 M^−2^ s^−1^ contained in the unreduced pseudo-first-order rate constant *k*′ in eqn (8). Table 1 summarises the kinetic parameters obtained. It can be seen that the presence of a small quantity of Ir^III^-complex promoter (for example, 1 mM) enhances the rate of the Q-Rh reduction reaction by a factor of 4; *i.e.* the promoting effect of Ir^III^-complex (1 mM) expressed as “enhancement ratio” is 
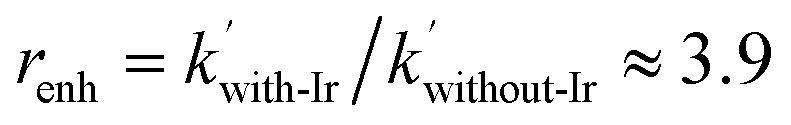
. Note that the enhancement ratio is independent of NADPH concentration and has the general formula shown in eqn (12).12
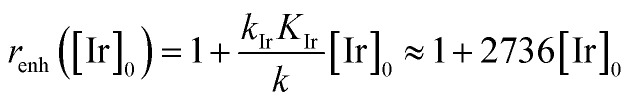
The enhancement ratio (plotted in Fig. 2b) can be used to determine how many times (*i.e. r*_enh_-times) faster is the Q-Rh reduction reaction in the presence of Ir^III^-complex promoter ([Ir]_0_ in the units of M) compared to the uncatalyzed (unpromoted) reaction in eqn (1). The individual constants in the product *k*_Ir_*K*_Ir_ cannot be extracted from the available kinetic data, although this does not preclude further analysis of the intracellular fluorescence behaviour of Q-Rh. It is clear that the non-zero *K*_Ir_ constant (*i.e.* NADPH·Ir complex formation in eqn (4)) plays a substantial role in the overall Q-Rh reduction kinetics. This is also emphasized by the relatively large value of the catalytic “boost” factor 
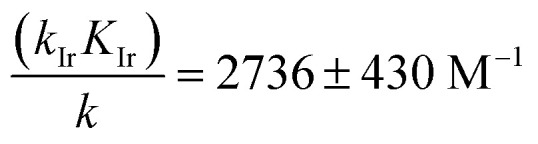
 in eqn (12), which highlights the activity of the Ir^III^-complex.

**Table 1:** Kinetic data (reaction rate constants) of Q-Rh reduction in PBS solution at 25 °C as obtained from time-dependent fluorescence measurements in Fig. 2a

[Ir]_0_ / mM	*k*′ / s^−1^^a^	Parameter^b^	Value
0.0	(2.8 ± 0.4) × 10^−4^	*k* / M^−1^ s^−1^	0.28 ± 0.04
0.5	(6.3 ± 0.2) × 10^−4^	*k*_Ir_ *K*_Ir_ / M^−2^ s^−1^	764 ± 85
1.0	(11.0 ± 0.5) × 10^−4^	*k*_Ir_ *K*_Ir_/*k* / M^−1^	2736 ± 430

a Value *k*′ as obtained from fitting of eqn (11).

b The values of *k*, *k*_M_*K*_M_, and the “boost” factor *k*_Ir_*K*_Ir_/*k* are obtained from the definition of pseudo-first-order rate constant *k*′ in eqn (8) using known concentrations of [NADPH]_0_ and [Ir]_0_.

The pseudo-first-order kinetics represented in eqn (7) was further tested by experiments where the initial concentration of NADPH was varied (at constant [Q-Rh]_0_ = 0.01 mM and [Ir]_0_ = 0.5 mM), with Q-Rh fluorescence emission monitored over time after the addition of Q-Rh to the solution at *t* = 0 min (Fig. 2c). The solid lines represent calculated (not fitted) behaviour as obtained using eqn (7) with *k* and *k*_Ir_*K*_Ir_ values taken from Table 1, [Q-Rh]_0_ = 0.01 mM, [NADPH]_0_ as a parameter (0–10 mM) and *t* as the independent variable. Data from Fig. 2c can also be used to generate the Q-Rh fluorescence decay as a function of NADPH concentration at a given constant time of 0–10 min (Fig. 2d). The solid lines again represent calculated (not fitted) behaviour using eqn (7) (with *k* and *k*_Ir_*K*_Ir_ from Table 1), [Q-Rh]_0_ = 0.01 mM, *t* as a parameter (0–10 min) and [NADPH]_0_ as the independent variable. It can be seen that the presented kinetic model describes well the experimental data.

Overall, from the above analyses, the kinetic model as introduced in eqn (1), (4) and (5), with the analytical solution represented by eqn (9) and (8) together with values in Table 1, yields a good description of the Q-Rh reduction process (*i.e.* formation of H_2_Q-Rh) and NADPH activation process (*i.e.* formation of NADP^+^) in the presence of Ir^III^-complex promoter in PBS solution (at pH = 7.4).

### 2.2 Kinetics at the interiors of HeLa cells

**Fig. 3:**
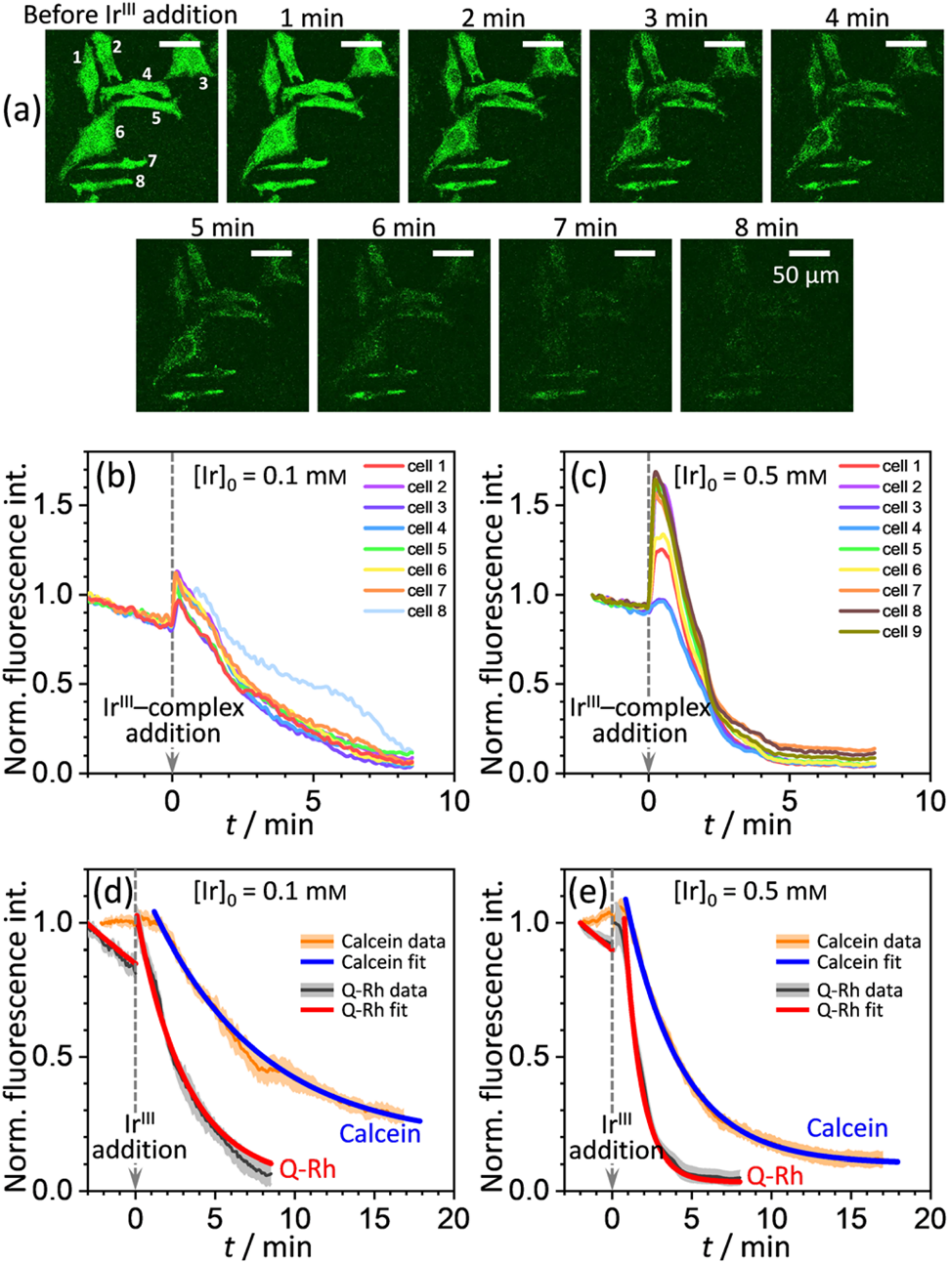
(a) Micrographs of fluorescence quenching of Q-Rh stained (0.01 mM) HeLa cells after addition of Ir^III^-complex (0.1 mM) at 25 °C. Normalized fluorescence time profiles from individual cells are shown in (b) and (c), and averaged data are shown in (d) and (e). (b) and (c) Plots of normalized fluorescence time profiles as obtained from individual HeLa cells (*λ*_ex_ = 488 nm, observed at 500–600 nm, 25 °C) stained with Q-Rh (0.01 mM) after the addition (at *t* = 0 min) of (b) 0.1 mM and (c) 0.5 mM of Ir^III^-complex. (d) and (e) Black lines show averaged and normalized Q-Rh fluorescence data from (b) and (c). Data are normalized prior to and following the addition of Ir^III^-complex (at *t* = 0 min). Orange lines show averaged and normalized fluorescence data of calcein stained (0.01 mM) HeLa cells after the addition (at *t* = 0 min) of (d) 0.1 mM and (e) 0.5 mM of Ir^III^-complex depicting the HeLa cell membrane disintegration (note that in this case, the Q-Rh is not present in HeLa cells). Grey and light orange backgrounds correspond to standard deviations. Blue and red lines are fitted according to eqn (14) and (16), respectively. Calcein data fitted parameters: (d) *k*_leak_ = (2.18 ± 0.34) × 10^−3^ s^−1^, *p* = 0.14 ± 0.03, *t*_0_ = 1.5 min; (e) *k*_leak_ = (4.82 ± 0.21) × 10^−3^ s^−1^, *p* = 0.10 ± 0.01, *t*_0_ = 1.2 min.

A series of experiments were performed using HeLa cells in order to analyze the intracellular kinetics of Q-Rh reduction with simultaneous NADPH activation. HeLa cells (with cellular passage number in the range 5–10) were incubated (30 min., 37 °C) with Q-Rh (0.01 mM) in Hanks' balanced salt solutions (HBSS) at pH = 7.4. Prior to fluorescence observation, HeLa cells placed in a glass bottom dish were rinsed twice with HBSS, and then the dish was filled with fresh HBSS. The average fluorescence intensity of each individual cell of the sample was obtained using a confocal laser scanning microscope on a cell culture maintained at 25 °C. Micrographs of Q-Rh fluorescence quenching in HeLa cells prior to and following the addition of Ir^III^-complex ([Ir]_0_ = 0.1 mM) are shown in Fig. 3a. Time dependence of the average fluorescence intensity as obtained from individual cells is plotted in Fig. 3b (for [Ir]_0_ = 0.1 mM) and Fig. 3c (for [Ir]_0_ = 0.5 mM). The sudden increase in fluorescence intensity following the addition of Ir^III^-complex is most likely due to injection shock (to some extent, an effect similar to addition of hypertonic solution and the corresponding change in the osmotic pressure). For further analyses, it is convenient to average and normalize the data before and after the addition of Ir^III^-complex, as shown in Fig. 3d and e (black lines). We have also observed that upon addition of Ir^III^-complex into the HeLa cell culture (in the absence of Q-Rh), the subsequent NADPH activation (or activation of other species) causes cell membrane disintegration, which is probably caused by the intracellular downstream signalling cascade (which regulates cell growth, proliferation, differentiation, and it can also trigger cell apoptosis^22^). We have used calcein acetoxymethyl (calcein-AM), a green fluorescent dye useful for cell viability monitoring, to stain the cytosol and then observed the dye leakage through HeLa cell membranes following the addition of Ir^III^-complex, as shown in Fig. 3d and e (orange lines) for addition of 0.1 mM and 0.5 mM of Ir^III^-complex, respectively. Due to its considerable effect, the leakage rate (*k*_leak_) has to be included in the overall Q-Rh reduction kinetics in HeLa cells. The leakage process was modelled as a release process in a confined space (*i.e.* the glass bottom dish), which is governed by the following differential equation.13
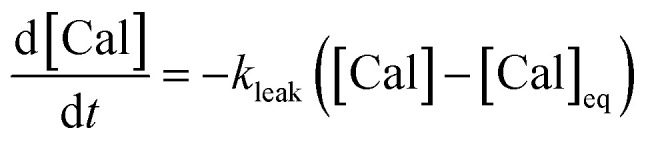
where [Cal] is the time-dependent concentration of calcein in the HeLa cells and [Cal]_eq_ (≠0) is the equilibrium calcein concentration at infinite time introduced due to confined space. The solution of eqn (13) can be expressed in terms of normalized intensity (similarly as for eqn (11)) as

**Fig. 4:**
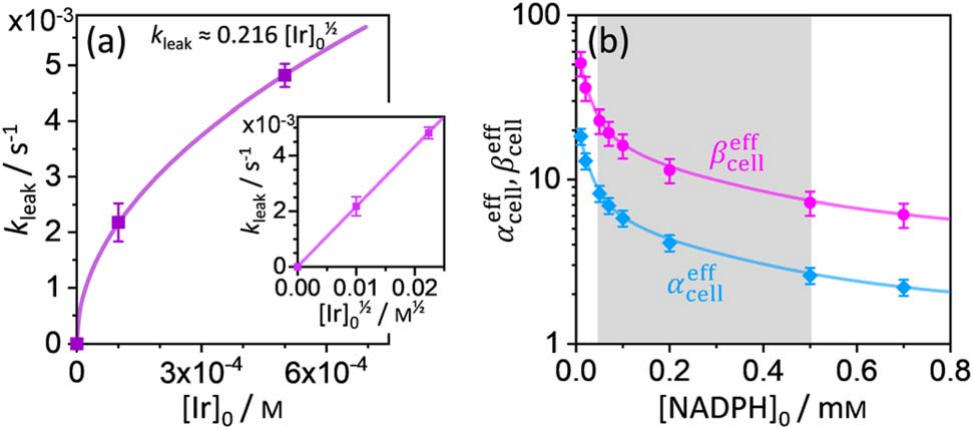
(a) Plot of leakage rate (*k*_leak_) against the concentration of added Ir^III^-complex [Ir]_0_ in HeLa cell cultures at 25 °C. The inset shows linear dependence of *k*_leak_ on [Ir]_0_^1/2^ indicating a power law dependence. (b) Effective rate increase coefficients *α*^eff^_cell_ and *β*^eff^_cell_ for uncatalyzed and Ir^III^-complex catalyzed Q-Rh reduction in HeLa cells, respectively, as a function of intracellular NADPH concentration. The grey region denotes HeLa cell available NADPH concentration range.

14 *I*_n,Cal_(*t*) = *p* + (1 − *p*)exp(−*k*_leak_(*t* − *t*_0_)),

where *p* = [Cal]_eq_/[Cal]_0_ is the fraction of remaining calcein fluorescence due to the confined space, and [Cal]_0_ is the initial calcein concentration at *t* = *t*_0_. Normalization means that *I*_n,Cal_(*t* = *t*_0_) = 1, where *t*_0_ can be interpreted as a lag time in fluorescence decrease after the addition of Ir^III^-complex in the HeLa cell culture. Eqn (14) is then fitted into experimentally observed calcein fluorescence data, as shown in Fig. 3d and e (blue lines; values of fitted parameters are shown in the caption). The leakage rate (*k*_leak_) has a power law type dependence on added Ir^III^-complex concentration *k*_leak_ = 0.216[Ir]_0_^1/2^, as shown in Fig. 4a (and the inset).

Using the above analysis of the HeLa cell membrane leakage rate after the addition of Ir^III^-complex, we can construct the overall pseudo-first-order rate constant for Q-Rh reduction kinetics inside the HeLa cells in the following form:15
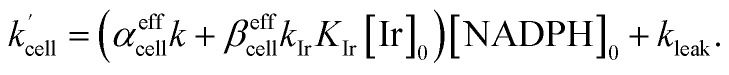
where *k*_leak_ can be well approximated as *k*_leak_ = 0.216[Ir]_0_^1/2^ (see Fig. 4a). The *α*^eff^_cell_ and *β*^eff^_cell_ are dimensionless coefficients, which account for the increase of the Q-Rh reduction rate inside HeLa cells due to the presence of other intracellular reduction agents, such as flavin adenine dinucleotide (FADH_2_),^23,24^ flavin mononucleotide (FMNH_2_)^16,23^ or glutathione (GSH).^25–28^

The *α*^eff^_cell_ and *β*^eff^_cell_ coefficients express effective rate increase of uncatalyzed Q-Rh reduction (shown in eqn (1)) and Ir^III^-complex catalyzed reduction (shown in eqn (5)), respectively, in HeLa cells as compared to the experiments in PBS solution (where of *α*^eff^_cell_ = *β*^eff^_cell_ = 1). It can also be noted that the *α*^eff^_cell_ and *β*^eff^_cell_ coefficients refer to the increase of Q-Rh reduction rate for *t* < 0 min and *t* > 0 min, respectively, as shown in Fig. 3d and e.

The rate constant 
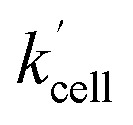
 is then used in eqn (16) (which is derived from eqn (7) analogously as eqn (11)) and fitted into normalized and averaged experimental data, as shown in Fig. 3d and e (red lines).16
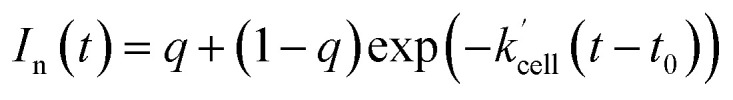
The *t*_0_ is a lag time in Q-Rh fluorescence decrease after the addition of Ir^III^-complex. In eqn (16)*α*^eff^_cell_, *β*^eff^_cell_ and *t*_0_ are fitted parameters. The 
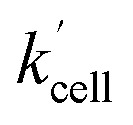
 rate constant (eqn (15)) contains [NADPH]_0_ as a known parameter, however, the NADPH concentration in the HeLa cells can range from about 0.05 mM to 0.5 mM (from here on, referred to as HeLa cell available NADPH concentration range).^29–33^ Therefore, the fitting of the experimental data in Fig. 3d and e (red lines) has been performed for various NADPH concentrations, and *α*^eff^_cell_ and *β*^eff^_cell_ coefficients were obtained as a function of [NADPH]_0_ (Fig. 4b). The data in Fig. 4b indicate that in the HeLa cell available NADPH concentration range (grey zone), the coefficients are within the following ranges: 2 < *α*^eff^_cell_ < 8 and 7 < *β*^eff^_cell_ < 23. From the fitting procedure, it can also be noted that in equilibrium, the Ir^III^-complex fluorescence quenching efficiency on Q-Rh seems to be the same (within the experimental error) as in PBS solution, *i.e.* 97%.

This large increase in reaction rates is due to the presence of the abovementioned reducing species in the cytosol, which apparently contribute to the Q-Rh reduction process before and, even more significantly, after the addition of Ir^III^-complex promoter. This can be quantified by the “enhancement ratio” *r*_enh,cell_, from which the component of leakage process has been removed, as17
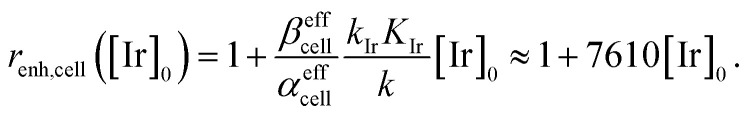
The ratio *β*^eff^_cell_/*α*^eff^_cell_ ≈ 2.8 over the whole NADPH admissible concentration region (Fig. 4b), which indicates an about 2.8 times higher Ir^III^-complex activity for the Q-Rh reduction process in HeLa cells over that in PBS solution (as seen by comparison with eqn (12)). Since the values of *α*^eff^_cell_ and *β*^eff^_cell_ are larger than 1 in the whole admissible region, it also suggests that other intracellular reducing agents besides NADPH (*e.g.* NADH, FADH_2_, FMNH_2_ or GSH) are also activated by the presence of the Q-Rh fluorescence probe alone (2 < *α*^eff^_cell_ < 8) and more strongly by the further addition of Ir^III^-complex (7 < *β*^eff^_cell_ < 23). It is worth mentioning that GSH is the main agent in the reduction process of intracellular quinones.^28^ The GSH contribution to the increase in Q-Rh reduction rate is probably dominant due to its high intracellular concentrations from 0.5 to 10 mM compared to other reducing agents.^25–28^ The high values of *α*^eff^_cell_ and *β*^eff^_cell_ coefficients (and accordingly increased 
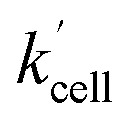
) most likely reflect this situation. There might also be a small contribution to the value of *α*^eff^_cell_ (and presumably also to the value of *β*^eff^_cell_) originating from the lower intracellular pH in HeLa cells (pH from *ca.* 5.7 to 7.1).^34–38^ Lower pH (less than 5.7) increases the reaction rate *k* in eqn (1).^39,40^ However, other research^21^ has shown that there is almost no effect on the reaction rate *k* in the pH range from 6 to 8. Therefore, there might be a limited contribution to the increase of the overall Q-Rh reduction rate constant in HeLa cells 
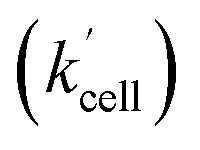
, which can be assigned to lower intracellular pH.

These results imply that the Q-Rh reduction process, which is accompanied by fluorescence quenching, can be used for the estimation of the relative rate of NADPH activation (*i.e.* the rate of NADP^+^ formation). The addition of Ir^III^-complex promoter can further increase this activation rate. In PBS solution, this process can be well controlled with an enhancement factor due to the Ir^III^-complex concentration expressed in eqn (12). In HeLa cells, the kinetics can also be analyzed, although the Q-Rh fluorescence quenching rate should be rather interpreted as the relative rate of activation involving several other intracellular reducing agents (not only NADPH). The presence of Ir^III^-complex also has a profound effect on the rate of the activation process, as expressed in eqn (17). Moreover, this activation caused by Ir^III^-complex inside the HeLa cells initiates a downstream signalling cascade, which results in cell membrane disintegration and cell death. This cascade is perhaps caused by the generation of reactive oxygen species, such as H_2_O_2_, which has been reported for similar organoiridium complex systems.^10,41,42^ The intrinsic cytotoxicity of the Ir^III^-complex is also evident from monitoring of calcein dye fluorescence after the addition of Ir^III^-complex (in the absence of Q-Rh), which initiates the cytosol leakage and eventual cell death. On the other hand, HeLa cells incubated only with Q-Rh (without the addition of Ir^III^-complex promoter) showed no signs of decreased cell viability.

## 3 Conclusions

We have shown that in the controlled environment of PBS solution, the fluorescent ubiquinone-rhodol (Q-Rh) probe reacts with NADPH leading to its quenched hydroquinone-rhodol (H_2_Q-Rh) form with simultaneous NADPH activation. This activation can be further increased by the addition of Ir^III^-complex (*i.e.* [(η^5^-C_5_Me_5_)Ir(phen)(H_2_O)]^2+^) promoter. The rate of Q-Rh fluorescence quenching process is proportional to NADPH activation rate. The kinetics of this process can be well-modelled by first-order kinetics for Q-Rh concentration with the pseudo-first-order rate constant involving the concentrations of Ir^III^-complex and NADPH.

Furthermore, we performed experiments on HeLa cells to analyze the intracellular kinetics of Q-Rh reduction and the influence of Ir^III^-complex promoter on the activation of intracellular reducing agents. We found that this process can also be modelled by modified first-order kinetics for Q-Rh. However, the Ir^III^-complex stimulates downstream intracellular processes, which result in HeLa cell membrane disintegration and leakage of the cytosol. Our kinetic model accounts for this process. Therefore, the actual fluorescence quenching of Q-Rh caused by reduction reactions can be quantified and their kinetic parameters extracted. There is a substantial increase in the Q-Rh reduction rate (accompanied by a corresponding increase of fluorescence quenching) inside the HeLa cells, especially after the addition of Ir^III^-complex promoter. This increase is partially due to the leakage process but also due to the nonspecific activation of other intracellular reducing agents other than NADPH, such as NADH, FADH_2_, FMNH_2_ or GSH (which might have the dominant contribution due to high intracellular GSH concentrations at mM levels). In the presence only of Q-Rh, the activation rate of the intracellular reducing agents is about 2 to 8 times greater in HeLa cells than in PBS solution. In the presence of both Q-Rh and Ir^III^-complex, the Ir^III^-complex catalyzed reduction reaction is about 7 to 23 times faster in HeLa cells.

The activation of NADPH or other intracellular species with simultaneous monitoring of this process can be used to exploit unique chemical reactions. This concept stands in contrast to the conventional, widely recognized concept of bioorthogonal chemistry. We have coined the term “bioparallel chemistry” to differentiate this approach. The analyses of Ir^III^-complex promoted NADPH activation, and its monitoring by Q-Rh fluorescence probe given in this study represents the first attempt to analyze the kinetics of a bioparallel reaction at the interiors of cells.

## 4 Experimental

### 4.1 General

Fluorescence spectra were measured on a JASCO FP-8500 spectrofluorophotometer using a quartz cuvette with a 1 cm path length. Phosphate-buffered saline (100 mM, pH 7.4) was used as a solvent. HeLa cells were obtained from RIKEN (Tsukuba, Japan), and cultured in Dulbecco's Modified Eagle Medium (DMEM) (Invitrogen, Carlsbad, CA, USA) containing 10% fetal bovine serum (FBS), 50 U mL^−1^ of penicillin and 50 μg mL^−1^ streptomycin at 37 °C under a humidified atmosphere of 5% CO_2_.

### 4.2 Time-dependent fluorescence measurements in PBS (Ir^III^-complex concentration dependence)

Time-dependent fluorescence intensity of Q-Rh (0.01 mM) reduction in PBS solution (at pH 7.4) was measured in the absence of the promoter (blank measurement), and with 0.5 mM and 1 mM of the Ir^III^-complex promoter, and with 1 mM NADPH which was added at time *t* = 0 min. During the measurements (with excitation wavelength *λ*_ex_ = 488 nm), the fluorescence was observed at its maximum at *λ*_em_ = 518 nm. Quenched fluorescence of Q-Rh reduced by sodium dithionite (Na_2_S_2_O_4_)^20^ to H_2_Q-Rh in PBS buffer was lowered to 1/30 of its original value (at 518 nm).^6^

### 4.3 Time-dependent fluorescence measurements in PBS (NADPH concentration dependence)

Time-dependence of fluorescence intensity during Q-Rh (0.01 mM) reduction was measured in PBS solution (at pH 7.4) with 0.5 mM of the Ir^III^-complex promoter following the addition (at *t* = 0 min) of various concentrations of NADPH (0, 0.25, 0.5, 1, 2.5, 5, and 10 mM). For these measurements, the excitation wavelength was *λ*_ex_ = 488 nm, and the fluorescence maximum was observed at *λ*_em_ = 519 nm.

### 4.4 Time-dependent fluorescence imaging of Q-Rh in HeLa cells

For fluorescent imaging experiments, a confocal laser scanning microscope system (FluoView FV1000; Olympus, Tokyo, Japan) mounted on an inverted microscope (IX81; Olympus) with a 40× or 60× oil-immersed objective lens was used. The fluorescence imaging measurements were performed on HeLa cells cultured on glass-bottomed dishes (Iwaki, Tokyo, Japan). For Q-Rh dye loading, the cells were incubated for 30 min at 37 °C in Hanks' balanced salt solutions (HBSS) containing NaCl (137 mM), KCl (5.4 mM), CaCl_2_ (1.3 mM), MgCl_2_ (0.5 mM), MgSO_4_ (0.4 mM), Na_2_HPO_4_ (0.3 mM), KH_2_PO_4_, (0.4 mM), NaHCO_3_ (4.2 mM), d-glucose (5.6 mM), HEPES (5.0 mM) (pH was adjusted to 7.4 using NaOH) in the presence of 0.01 mM Q-Rh. HeLa cells were washed twice with HBSS solution to remove the remaining extracellular dye, and fluorescence imaging measurements were subsequently performed. Q-Rh was excited at *λ*_ex_ = 488 nm, with the signal being observed at 500–600 nm. Fluorescence images were acquired and processed in the FluoView software package (Olympus). The fluorescence intensities were determined by calculating the average intensity within a defined region of interest that encompassed the cell body of each cell. Fluorescence imaging indicates that Q-Rh is uniformly distributed in the cytosol with partial accumulation in mitochondria.

## Conflicts of interest

There are no conflicts to declare.

## Supplementary Material
